# Examining the effects of femoral anteversion and passive hip rotation on ACL injury and knee biomechanics: a systematic review and meta-analysis

**DOI:** 10.1186/s40634-022-00479-7

**Published:** 2022-05-05

**Authors:** Jennifer A. Hogg, Justin P. Waxman, Sandra J. Shultz

**Affiliations:** 1 Department of Health and Human Performance, The University of Tennessee Chattanooga, 615 McCallie Ave, Chattanooga, TN 37403 USA; 2 Biomechanics Division, S-E-A, Ltd, Columbus, OH USA; 3 Department of Kinesiology, The University of North Carolina at Greensboro, Greensboro, NC USA

**Keywords:** Sex-specific, Valgus, Frontal plane knee projection angle, Sport injury

## Abstract

**Purpose:**

Greater femoral internal rotation (via anteversion or passive hip ROM) is associated with knee biomechanics thought to contribute to anterior cruciate ligament (ACL) injury, but it is unknown if femoral internal rotation contributes to actual ACL injury occurrence. The objective of this systematic review and meta-analysis was to quantify the extent to which femoral anteversion and hip range of motion (ROM) influence knee biomechanics consistent with ACL injury and actual ACL injury occurrence.

**Methods:**

Using PRISMA guidelines, PubMed, CINAHL, SportDiscus, and Scopus databases were searched. Inclusion criteria were available passive hip ROM or femoral anteversion measure, ACL injury OR biomechanical analysis of functional task. Two reviewers independently reviewed titles, abstracts, and full texts when warranted. Included studies were submitted to Downs & Black Quality Assessment Tool. Meta-analyses were conducted for comparisons including at least two studies.

**Results:**

Twenty-three studies were included (11 injury outcome, 12 biomechanical outcome). Decreased internal rotation ROM was significantly associated with history of ACL injury (MD -5.02°; 95% CI [-8.77°—-1.27°]; *p* = 0.01; *n* = 10). There was no significant effect between passive external rotation and ACL injury (MD -2.62°; 95% CI [-5.66°—- 0.41°]; *p* = 0.09; *n* = 9) Participants displaying greater frontal plane knee projection angle had greater passive external rotation (MD 4.77°; 95% CI [1.17° – 8.37°]; *p* = 0.01; *n* = 3). There was no significant effect between femoral anteversion and ACL injury (MD -0.46°; 95% CI [-2.23°—1.31°]; *p* = 0.61; *n* = 2). No within-sex differences were observed between injured and uninjured males and females (*p* range = 0.09 – 0.63).

**Conclusion:**

Though individuals with injured ACLs have statistically less passive internal and external rotation, the observed heterogeneity precludes generalizability. There is no evidence that femoral anteversion influences biomechanics or ACL injury. Well-designed studies using reliable methods are needed to investigate biomechanical patterns associated with more extreme ROM values within each sex, and their prospective associations with ACL injury.

**Level of evidence:** IV.

**Supplementary Information:**

The online version contains supplementary material available at 10.1186/s40634-022-00479-7.

## Introduction

Of the more than 150,000 ACL injuries that occur annually in the United States, 70% occur through non-contact mechanisms [7, 8]. These mechanisms have been studied extensively both in vivo and in vitro [2, 5], and have led to the idea that functional valgus collapse, a multiplanar mechanism, may put one at risk of ACL injury [42]. The combination of hip adduction and internal rotation and tibial abduction and external rotation mechanism was first proposed as an ACL injury mechanism by Ireland [24] and subsequently documented in retrospective videographic studies [8, 29]. Compared with sex-matched controls, ACL-injured subjects moved progressively into greater dynamic knee valgus, with the injured cohort displaying frontal plane knee angles ten degrees greater than uninjured controls at the assumed moment of injury (30-50 ms after initial contact) [8, 18, 30]. Moreover, females are reported to display this valgus collapse injury mechanism more frequently than males [29].

As ACL injury mechanisms are becoming better understood, attention has turned toward factors precipitating the poor knee biomechanics that may contribute to these mechanisms. It is accepted that movement occurs in a proximal-to-distal fashion [14, 27], particularly in an open-kinetic chain system, where movement is generated proximally and transferred distally. Because ACL injuries occur so quickly following initial ground contact (30-50 ms) [30] it is possible that proximal factors also influence closed-chain ACL injury mechanisms as the literature suggests that aberrant hip kinematics may contribute to poor knee biomechanics [21, 25, 46]. Because the hip and knee are coupled joints, knee abduction and rotation may follow hip adduction and hip rotation, respectively, when the foot is fixed [23]. Thus, dynamically controlling the hip may be crucial to improved knee control.

Two characteristics thought to influence dynamic hip adduction and internal rotation are femoral anteversion and hip range of motion (ROM) [21, 38]. Specifically, increased femoral anteversion and greater internal rotation ROM are suggested to bias the femur toward internal rotation and adduction at the hip, which may predispose one to greater valgus collapse at the knee [39, 40, 46]. Consistent with this theory, females are known to have greater amounts of both femoral anteversion and hip internal rotation ROM [37, 41] as well as greater prevalence of valgus collapse. Moreover, retrospective evidence suggests a link between increased femoral anteversion/hip ROM and ACL injury status [16, 48]. It is unknown whether femoral anteversion and passive hip ROM influence biomechanics and ACL injury status in a consistent manner (i.e. a femur biased to internal rotation is associated with greater valgus AND higher ACL injury risk). In other words, is valgus collapse the mechanistic connection between femoral orientation and ACL injury status, and are these relationships different between sexes? Understanding the underlying risk factors that promote *both* high-risk biomechanics and ACL injury are important for informing neuromuscular training strategies and determining proximal characteristics may ultimately influence ACL injury risk in men and women.

The objective of this systematic review was to pool relevant literature and quantify the extent to which femoral anteversion and hip ROM influence knee biomechanics, specifically measures of functional valgus, and ACL injury. Specifically, the following questions were of interest: 1) Do femoral anteversion and hip ROM impact ACL injury occurrence? 2) Do femoral anteversion and hip ROM impact knee biomechanics? 3) Is there evidence to support a sex-specific injury mechanism? (i.e., Are the relationships between femoral anteversion and hip ROM with knee biomechanics and injury outcomes different in males and females?). We hypothesized that greater femoral anteversion and hip internal rotation and lesser external rotation will be associated with greater functional valgus collapse and with a higher incidence of ACL injury. Additionally, we hypothesized that both effects would be greater in female cohorts. Answering these questions is necessary to determine if femoral anteversion and hip ROM contribute to ACL injury through altering biomechanics, and if risk factors are the same for males and females.

## Methods

### Study design

This systematic review was conducted according to PRISMA guidelines [34]. Because there was no direct interaction with participants, Institutional Review Board approval was unnecessary. This systematic review did not use a registered protocol and was not supported by any external funding.

### Search strategy

An initial electronic search of PubMed, CINAHL, SPORTDiscus, and Scopus was conducted on April 21, 2016, and a follow-up search was conducted on May 5, 2021. No restrictions were placed on the date of publication, nor were any initial restrictions placed on language. The following search strategy was used: (anterior cruciate ligament OR ACL) AND hip AND (range of motion OR rotation OR anteversion).

### Eligibility criteria

Studies were included in this systematic review if they met the following criteria: 1) the study design was prospective, retrospective or cross-sectional, 2) the sample cohort consisted of athletes or healthy participants, 3) detailed methods of hip ROM and/or femoral anteversion were reported, such that it would be easily replicable, 4) means and standard deviations for hip ROM and/or femoral anteversion were provided, or sufficient data were available to compute an effect size, 5) participants with ACL injury had their injury verified surgically or via MRI, and 6) participants undergoing biomechanical assessment must have had 3D or 2D knee and hip biomechanical evaluation during a functional task (squatting, cutting, or landing) were reported. Prospective and retrospective studies were considered appropriate for determining the relationship between femoral anteversion, hip ROM, and ACL injury. Cross-sectional studies were considered appropriate for determining the relationship between femoral anteversion, hip ROM, and biomechanics. Thus, both types of studies were considered for inclusion. If a study was identified that appeared to fit the above criteria, but key data were missing (e.g., sex-stratified descriptive data), the author was contacted for the necessary information.

### Study selection

Compilation and management of all retrieved articles were conducted using Excel Workbooks for Systematic Reviews (Helena VonVille, University of Texas School of Public Health Library, 2015). The initial and final searches were conducted by the primary author (JAH) on April 21, 2016 and May 5, 2021, respectively. After duplicates were removed, all titles and abstracts were independently screened for initial inclusion by two reviewers (JAH & JPW). Discrepancies between the two reviewers were settled by consensus. If no consensus could be reached, a third reviewer (SJS) was consulted. All remaining articles then underwent full-text review by both reviewers. Each remaining article was read in its entirety, and final eligibility was determined based on the above criteria. Reference lists of all included studies were hand-searched by two reviewers (JAH & JPW) to identify additional studies fitting inclusion criteria. Cohen’s Kappa was calculated after initial review and before reconciliation of differences to determine inter-reviewer agreement.

### Quality assessment

The quality of each included study was examined using the Quality Index developed by Downs & Black [13] for the assessment of non-randomized studies. The original checklist consists of 27 items; however, not all 27 items were applicable to the chosen study design. Thus, a modified 14 item checklist was used, broken into four subscales: Reporting, External Validity, Internal Validity-Bias, and Internal Validity-Confounding (Table 1). The original checklist was reported to have a Spearman’s correlation coefficient of 0.79 for test–retest reliability and 0.75 for inter-rater reliability [13]. Reported kappa values for test–retest reliability of the items included on the modified checklist ranged from 0.35–1.00, with a mean of 0.65. One reviewer (JAH) assessed the quality of each included study.

**Table 1:** Modified Downs & Black Quality Index

Reporting
Q1. Is the hypothesis/objective of the study clearly described?
Q2. Are the main outcomes to be measured clearly described in the Introduction or Methods?
Q3. Are the characteristics of the patients included in the study clearly described?
Q5. Are the distributions of principal confounder in each group of subjects to be compared clearly described?
Q6. Are the main findings of the study clearly described?
Q7. Does the study provide estimates of the random variability in the data for the main outcomes?
Q10. Have actual probability values been reported (e.g. 0.035 rather than < 0.05) for the main outcomes except where the probability value is less than 0.001?
**External Validity**
Q11. Were the subjects asked to participate in the study representative of the entire population from which they were recruited?
**Internal Validity-Bias**
Q15. Was an attempt made to blind those measuring the main outcomes of the intervention? (only for retrospective studies)
Q16. If any of the results of the study were based on “data dredging”, was this made clear?
Q18. Were the statistical tests used to assess the main outcomes appropriate?
Q20. Were the main outcome measures used accurate (valid and reliable)?
**Internal Validity-Confounding**
Q21. Were the patients in different intervention groups (trials and cohort studies) or were the cases and controls (case–control studies) recruited from the same population? (only for retrospective studies)
Q27. Did the study have sufficient power to detect a clinically important effect where the probability value for a difference being due to chance is less than 5%?

*Adapted from Downs & Black, 1998

### Data extraction

The following data were extracted from every included study: participant activity level, participant age, descriptive statistics (mean ± SD) of femoral anteversion and/or hip ROM, reliability (if available), and effect size (Cohen’s d). For studies in which ACL injury was the outcome, the amount of time elapsed between ACL injury and clinical measurement was also extracted. For biomechanical studies, the functional task used and its associated kinematic or kinetic outcome variables were extracted. For all included studies, if no effect size was reported, then Cohen’s d was calculated between ACL-injured and healthy groups, or between high-risk and low-risk groups. If the effect size was reported as an R^2^ value, it was re-expressed as a Cohen’s d for more ready comparison. If reported data were insufficient to calculate an effect size, the author was contacted for further information [6, 9, 11]. Meta-analyses were performed for all between-group comparisons (i.e., ACL-injured v. healthy, displaying medial knee displacement v. no medial knee displacement) containing at least two studies.

## Results

### Search results

**Fig. 1:**
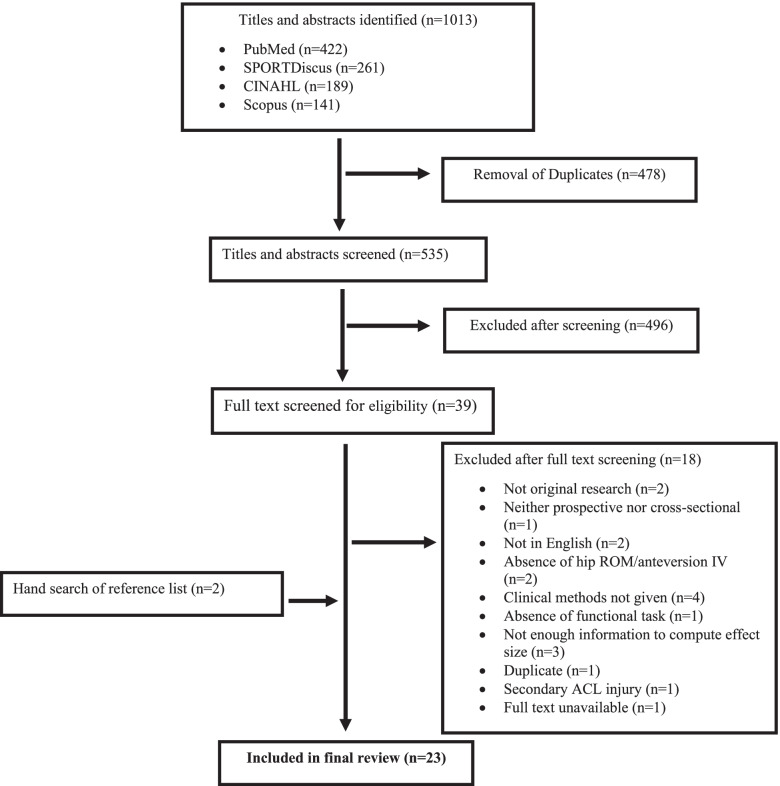
Flow diagram depicting study selection

An electronic database search of PubMed, SPORTDiscus, CINAHL, and Scopus yielded 1,013 titles and abstracts. After 478 duplicates were removed, the remaining 535 titles and abstracts were screened by two reviewers (JAH & JPW) for initial eligibility (Cohen’s κ = 0.87, 95% CI 0.76–0.98). After which, two reviewers read the full-text of 39 articles to determine eligibility for final inclusion. Of the 39 studies reviewed, 18 were excluded, leaving 21 studies. The reference lists of the remaining studies were inspected for relevant articles, of which two additional articles were found. Thus, a total of 23 studies were included in the final review (Fig. 1).

### Study characteristics

Of the 23 studies included in this review, eleven were of a retrospective design reporting ACL injury and twelve were cross-sectional studies assessing lower extremity biomechanics. General characteristics of these studies are presented in Tables 2 and 3.

**Table 2:** Characteristics of included injury studies

Study (year)	Participants	Age of Participants (yrs)	Time Elapsed Post Injury	Level of Evidence
Bagherifard et al. (2018) [1]	127 non-professional athletes with ACL injury (13F, 113 M), 90 (11F, 79 M) non-ACL injured patients	27.8 ± 6.1 and 28.9 ± 6.3, respectively	Not reported	Level III
Bedi et al. (2016) [3]	34 injured National Football League players; 290 uninjured National Football League players	Not reported	Not reported	Level IV
Budinski et al. (2016) [11]	60 active ACL-injured males (uninjured limb control)	24.86 (range 15–46 years)	< 6 months	Level III
Daneshmandi et al. (2012) [12]	20 injured females, 20 uninjured females	24.9 ± 5.8 and 24.8 ± 5.6, respectively	2 years	Level III
Gomes et al. (2008) [16]	50 injured male soccer athletes, 50 injured male soccer controls	28.1 ± 5.7 and 23.3 ± 5.4, respectively	Not reported	Level III
Hertel et al. (2004) [17]	20 ACL injured (10F, 10 M), 20 uninjured controls (10F, 10 M)	20.7 ± 1.4 and 20.4 ± 1.2, respectively	3–84 months	Level III
Kramer et al. (2007) [28]	33 ACL injured females, 33 female controls	21 ± 2.1 and 19.6 ± 1.3, respectively	5 years	Level III
Lopes et al. (2016) [31]	45 non-contact ACL injured males, 35 contact ACL injured males	Aged 18–40	< 6 months	Level III
Lopes et al. (2017) [32]	41 male ACL-injured patients, 39 male uninjured patients	Aged 20–40	Not reported	Level III
Tainaka et al. (2014) [48]	44 ACL injured (21F, 23 M), 123 healthy controls (49F, 74 M)	Aged 13–17	Several weeks	Level III
VandenBerg et al. (2017) [49]	25 ACL-injured (12F, 13 M) and 25 control patients (12F, 13 M)	22.8 ± 7.2 and 24.5 ± 7.9, respectively	Within 3 months post-injury	Level III

Note: *M* male; *F* female

**Table 3:** Characteristics of included biomechanical studies

Study (year)	Participants	Age of Participants (yrs)	Level of Evidence
Bell et al. (2008) [4]	37 healthy participants (15F, 4 M)	20.7 ± 2.1	Level IV
Bittencourt et al. (2012) [6]	254 athletes (79F, 175 M)	16.6 ± 5.0	Level IV
Breen et al. (2010) [9]	16 participants (8F, 8 M)	21 ± 3	Level IV
Hogg et al. (2019) [20]	20 healthy participants (20F)	24.9 ± 4.1	Level IV
Hogg et al. (2021) [19]	90 healthy participants (45F, 45 M)	20.5 ± 1.9	Level IV
Howard et al. (2011) [21]	45 healthy participants (30F, 15 M)	21 ± 2	Level IV
Kaneko et al. (2013) [26]	16 healthy females	20.8 ± 1.0	Level IV
Mauntel et al. (2013) [35]	20 healthy females and 20 males	20.2 ± 1.7	Level IV
Nguyen et al. (2015) [40]	141 active adults (91F, 50 M)	21.7 ± 2.7	Level IV
Nguyen et al. (2011) [38]	60 healthy adults (30F, 30 M)	23.1 ± 3.1	Level IV
Rabin & Kozol (2010) [44]	29 healthy females	24.3 ± 3.2	Level IV
Stiffler et al. (2015) [47]	27 active subjects with medial knee displacement (21F, 6 M), 70 controls (48F, 22 M)	20.2 ± 1.4 and 20.3 ± 1.5, respectively	Level IV

Note: *M* male; *F* female

#### Studies with ACL injury as the outcome

All participants in the retrospective injury risk studies were considered recreationally active. All studies except for one [31] were predominantly made up of participants under age 30, with two [31, 32] including participants up to age 40. Thus, the ages of the sample populations across studies were rather homogenous. Male participants across studies numbered 979, in contrast to 244 females. The time elapsed between ACL injury occurrence and data collection varied widely between studies, ranging from a few weeks up to five years. Four studies [16, 32, 42] did not report time elapsed between injury occurrence and data collection.

Quality assessment scores for each study are detailed in Table 4. The average Downs & Black Quality Index score in the retrospective injury risk studies was 9.5/14 (range = 8–13). The most commonly neglected items were blinding of the investigator and reporting of statistical power.

**Table 4:** Downs & Black Quality Index Scores for included studies

	**Reporting**							**External Validity**	**Internal Validity-Bias**				**Internal Validity-Confounding**		
*no* = *0, yes* = *1*	Q1	Q2	Q3	Q5	Q6	Q7	Q10	Q11	Q15	Q16	Q18	Q20	Q21	Q27	**Total**
Injury outcome															
**Bagherifard(2018) **[1]	0	1	1	1	1	1	0	1	0	0	1	0	1	0	**8**
**Budinski(2016) **[11]	0	1	0	1	1	1	1	1	0	1	0	0	1	0	**8**
**Bedi(2016) **[3]	1	1	1	1	0	0	1	1	0	1	1	0	1	0	**9**
**Daneshmandi(2012) **[12]	0	1	1	1	1	1	1	1	0	1	1	1	0	0	**10**
**Gomes(2008) **[16]	1	1	1	0	1	1	1	1	0	0	1	0	0	0	**8**
**Hertel(2004)** [17]	1	1	1	1	1	1	1	1	0	1	1	1	1	0	**12**
**Kramer(2007)** [28]	0	1	1	1	1	1	1	1	0	1	1	0	0	0	**9**
**Lopes(2016) **[31]	1	1	1	0	1	1	1	1	1	0	0	0	0	1	**9**
**Lopes(2017) **[32]	0	1	0	0	1	1	1	1	1	1	1	0	0	0	**8**
**Tainaka(2014) **[48]	0	1	1	1	1	1	1	1	0	1	1	1	1	0	**11**
**VandenBerg(2017) **[49]	1	1	1	1	1	1	1	1	0	1	1	1	1	1	**13**
Biomechanic outcome															
**Bell(2008) **[4]	1	1	1	1	1	1	1	1	NA	1	1	1	NA	1	**12**
**Bittencourt(2012) **[6]	0	1	1	1	1	1	1	1	NA	1	1	1	NA	0	**10**
**Breen(2010) **[9]	1	1	0	1	1	0	1	1	NA	1	0	0	NA	1	**8**
**Hogg(2019) **[20]	1	1	1	1	1	1	1	1	NA	1	1	1	NA	0	**11**
**Hogg (2021)**[19]	1	1	1	1	1	1	1	1	NA	1	1	1	NA	1	**12**
**Howard(2011) **[21]	1	1	1	1	1	1	1	1	NA	1	1	1	NA	1	**12**
**Kaneko(2013) **[26]	1	1	1	1	1	1	0	1	NA	1	0	0	NA	0	**8**
**Mauntel(2013) **[35]	1	1	1	1	1	1	1	1	NA	1	1	1	NA	1	**12**
**Nguyen(2015) **[40]	1	1	1	1	1	1	1	1	NA	1	1	1	NA	0	**11**
**Nguyen(2011) **[38]	1	1	1	1	1	1	0	1	NA	1	1	1	NA	0	**10**
**Rabin(2010) **[44]	1	1	1	1	1	1	0	1	NA	1	1	1	NA	0	**10**
**Stiffler(2015) **[47]	1	1	1	1	1	1	1	1	NA	1	1	0	NA	0	**10**

**Fig. 2:**
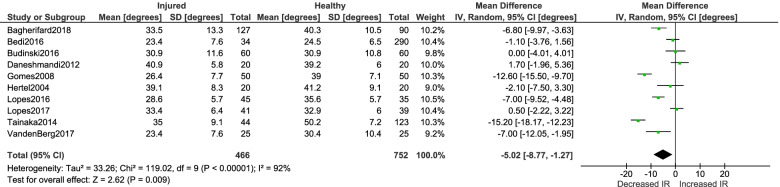
Meta-analysis results detailing the association between passive internal rotation ROM and ACL injury

**Fig. 3:**
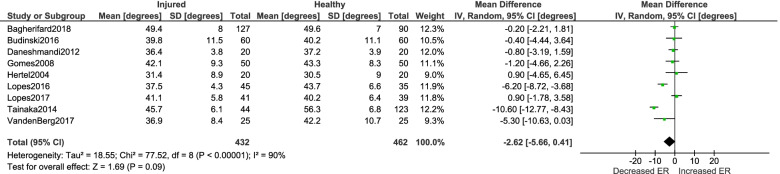
Meta-analysis results detailing the association between passive external rotation ROM and ACL injury

**Fig. 4:**
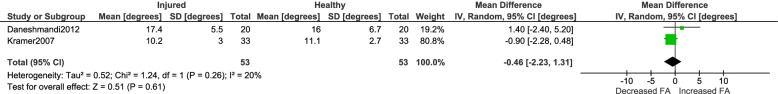
Meta-analysis results detailing the association between femoral anteversion and ACL injury

Detailed results of each injury risk study are presented in Table 5. Meta-analyses were conducted for the injury risk studies to assess aggregate between-group differences in femoral anteversion, passive internal rotation and external rotation between injured limbs and healthy control limbs. Fewer degrees of passive internal rotation was significantly associated with history of ACL injury based on the compiled data of 10 studies (MD -5.02°; 95% CI [-8.77°—-1.27°]; *p* = 0.01; *n* = 10) (Fig. 2). There was no significant effect between passive external rotation and ACL injury when examining the metadata of nine studies (MD -2.62°; 95% CI [-5.66° – 0.41°]; *p* = 0.09; *n* = 9) (Fig. 3). A meta-analysis of two studies, both in females, revealed no significant effect between femoral anteversion and ACL injury (MD -0.46°; 95% CI [-2.23° – 1.31°]; *p* = 0.61; *n* = 2) (Fig. 4).

**Table 5:** Results of studies examining the relationship between transverse plane femoral alignment and history of ACL injury

Study (year)	Clinical Measure(s)	Clinical Measure(s) Mean (SD) (injured, control)	Reliability of Clinical Measure	*P* value	Effect size (Cohen’s *d*)
Bagherifard et al. (2018) [1]	Internal rotation ROM External rotation ROM	33.5(13.3), 40.3(10.5) 49.4(8.0), 49.6(7.0)	Not reported	< .001 > .05	0.6 0.0
Budinski et al. (2016) [11]	Internal rotation ROM External rotation ROM	30.9(11.6), 30.9(10.8) 39.8(11.5), 40.2(11.1)	Not reported	> .99 .85	0.0 0.0
Bedi et al. (2016) [3]	Internal rotation ROM	23.4(7.6), 24.5(6.5)	Not reported	.36	0.2
Daneshmandi et al. (2012) [12]	Femoral anteversion Internal rotation ROM External rotation ROM	17.4(5.5), 16.0(6.7) 40.9(5.8), 39.2(6.0) 36.4(3.8), 37.2(3.9)	ICC > .85 (SEM not reported)	.46 .38 .51	0.2 0.3 0.2
Gomes et al. (2008) [16]	Internal rotation ROM External rotation ROM	26.4(7.7), 39.0(7.1) 42.1(9.3), 43.3(8.3)	Not reported	.001 .48	1.7 0.1
Hertel et al. (2004) [17]	Internal rotation ROM External rotation ROM	39.1(8.3), 41.2(9.1) 31.4(8.9), 30.5(9.0)	ICC > .70 (SEM not reported)	.30 .35	0.2 0.1
Kramer et al. (2007) [28]	Femoral anteversion	10.2(3.0), 11.1(2.7)	Not reported	.06	0.3
Lopes et al. (2016) [31]	Internal rotation ROM External rotation ROM	28.6(5.7), 35.6(5.7) 37.5(4.3), 43.7(6.6)	Not reported	.001 .001	1.2 1.1
Lopes et al. (2017) [32]	Internal rotation ROM External rotation ROM	33.4(6.4), 32.9(6.0) 41.1(5.8), 40.2(6.4)	ICC = 0.98 (SEM not reported)	.67 .50	0.1 0.1
Tainaka et al. (2014) [48]	Internal rotation ROM External rotation ROM	35.0(9.1), 50.2(7.2) 45.7(6.1), 56.3(6.8)	Test–retest Pearson *R* > .85	.0001 .0001	1.9 1.6
VandenBerg et al. (2017) [49]	Internal rotation ROM External rotation ROM	23.4(7.6), 30.4(10.4) 36.9(8.4), 42.2(10.7)	ICC = 0.90 (SEM not reported)	.01 .06	0.8 0.6

Heterogeneity within the retrospective ACL injury studies was highly variable; I^2^ ranged from 20% for the influence of femoral anteversion on ACL injury to 90% and 92% for the influence of internal and external rotation on ACL injury, respectively.

#### Cross-sectional studies where lower extremity biomechanics was the outcome

With one exception (not reported) [9], all participants in the biomechanical studies were deemed physically active, and all were below the age of 30. Unlike the injury studies, which were composed mostly of males, the biomechanical studies included 452 females and 375 males. Of the twelve studies, eleven used a squat or jump landing variation to determine the influence of femoral anteversion and passive hip rotation on biomechanics. Of these eleven, three used a double-leg task [4, 40, 47] and eight opted for a single-leg task [6, 19–21, 26, 35, 38, 44]. A single study chose a side-cutting task [9]. Seven of the studies measured valgus motion via 3D biomechanics [9, 19–21, 26, 38, 40]; the remaining five [4, 6, 35, 44, 47] measured 2D frontal plane knee motion.

The average Downs & Black score for the cross-sectional biomechanical studies was 10.5/12 (range = 8–12). Though the majority of these studies also omitted mention of statistical power, they generally scored higher on the Quality Index scale than did the retrospective injury risk studies. Because 2D biomechanics represent different movement patterns than 3D mechanics, they are reported separately below.

##### Biomechanical 2D results

**Fig. 5:**
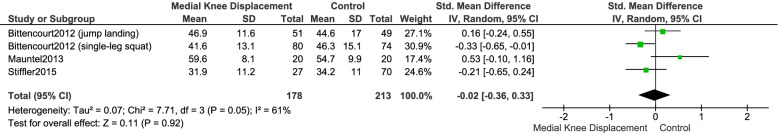
Meta-analysis results detailing the association between internal rotation ROM and 2D frontal plane knee projection angle

**Fig. 6:**
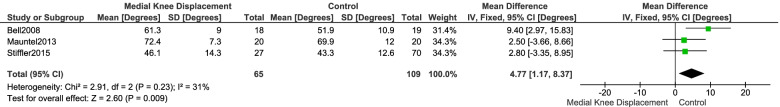
Meta-analysis results detailing the association between external rotation ROM and 2D frontal plane knee projection angle

Detailed results of each biomechanical study are presented in Table 6. Of the seven studies assessing 2D biomechanics, three studies [6, 35, 47] examined the association between femoral anteversion and passive internal rotation and 2D medial knee displacement (knee abduction) using single-leg squats [6, 35] or double-leg jump landings [47] (Figs. 5 and 6). Only one study [6] detected a small effect (*d* = 0.3; *p* = 0.04) during a single-leg squat between groups displaying high and low internal rotation ROM. Three studies [4, 35, 47] examined the association between passive external rotation and 2D medial knee displacement using an overhead squat [4], a single-leg squat [35], and a double-leg jump landing [47] (Fig. 6). All three studies observed greater passive external rotation in participants with medial knee displacement (collectively, participants with greater external rotation ROM displayed 4.77° greater two-dimensional frontal plane knee angle), though Bell (2008) [12] was the only study in which the external rotation difference was significant (MD 9.40°; CI [2.97 – 15.83]; *p* = 0.01).

**Table 6:** Results of studies examining the relationship between transverse plane femoral alignment and biomechanics during a functional task

Study (year)	Clinical Measure(s)	Clinical Measure(s) Mean (SD) (high risk, low risk (if applicable)	Reliability of Clinical Measure (ICC(SEM)	Functional Task	Outcome Measure(s)	*P* value	Effect size (Cohen’s *d*)
Bell et al. (2008) [4]	External rotation ROM	61.3(9.0), 51.9(10.9)	.87-.96 (1.6–2.6°)	Overhead squat	2D medial knee displacement	.01	0.9
Bittencourt et al. (2012) [6]	Internal rotation ROM	41.6(13.1), 46.3(15.1) 46.9(11.6), 44.6(17.0)	.99 (1.5°)	Single leg squat and jump landing	Frontal plane projection angle	.04 .43	0.3 0.2
Breen et al. (2010) [9]	Internal rotation ROM External rotation ROM	30.29(8.8), 44.46(6.0) 43.21(8.7), 46.54(8.0)	Not reported	Maximal drop jump with diagonal side cut	Knee flexion at initial contact Hip flexion at initial contact Dorsiflexion at initial contact Thigh rotation at initial contact Shank rotation at initial contact	.003 .87 .31 .32 .14	1.8 0.1 0.6 0.5 0.8
Hogg et al (2019) [20]	Internal rotation ROM External rotation ROM Femoral anteversion	31.3 ± 8.4 44.6 ± 9.8 10.3 ± 5.8	.97(1.6) .85(3.3) .92(1.2)	Single-leg forward landing	Peak hip adduction moment	.63 .75 .91	0.8 0.2 0.1
					Peak hip internal rotation moment	.10 .85 .07	0.8 0.1 0.9
					Peak knee abduction moment	.05 .95 .57	1.0 0.0 0.3
					Peak knee internal rotation moment	.16 .09 .92	0.7 0.9 0.0
					Peak hip adduction angle	.47 .78 .88	0.3 0.1 0.1
					Peak hip internal rotation angle	.40 .70 .13	0.4 0.2 0.7
					Peak knee abduction angle	.63 .81 .87	0.2 0.1 0.1
					Peak knee internal rotation angle	.68 .36 .86	0.2 0.4 0.1
Hogg et al. (2021) [19]	Internal rotation ROM External rotation ROM Femoral anteversion		.97(1.6) .85(3.3) .92(1.2)	Single-leg forward landing	Peak hip adduction moment	.07 .77 .02	0.4 0.1 0.5
					Peak hip internal rotation moment	.01 .10 .32	0.6 0.4 0.2
					Peak knee abduction moment	.12 .95 .80	0.3 0.0 0.1
					Peak knee internal rotation moment	.66 .29 .86	0.1 0.2 0.0
					Peak hip adduction angle	.11 .69 .08	0.3 0.1 0.4
					Peak hip internal rotation angle	.002 .01 .01	0.7 0.6 0.6
					Peak knee abduction angle	.001 .89 .05	0.7 0.0 0.4
					Peak knee internal rotation angle	.43 .05 .32	0.2 0.4 0.2
Howard et al. (2011) [21]	Internal rotation ROM External rotation ROM	29.0(11.0) 35.0(7.0)	.99(.5°) .95(1.9°)	Single-leg jump landing	Hip adduction excursion	.02	0.8
					Knee abduction excursion	.001	1.2
					Knee adduction excursion	.03	0.7
					Knee external rotation excursion	.005	0.9
Kaneko et al. (2013) [26]	Femoral anteversion	20.7(3.3), 16.1(1.7)	Not reported	Single-leg jump landing	Hip flexion at IC and 100 ms PC	.01, .01	1.6, 1.7
					Hip abduction at IC and peak	.38, .10	0.5, 1.1
					Knee flexion at IC and peak	.42, .04	0.5, 1.4
					Knee valgus at IC and peak	1.00, .03	0.0, 1.5
Mauntel et al. (2013) [35]	Internal rotation ROM External rotation ROM Femoral anteversion	59.6(8.1), 54.7(9.9) 72.4(7.3), 69.9(12.0) 3.2(2.8), 3.5(3.1)	.89(4.5°) .64(7.5°) .73(0.9°)	Single-leg squat	Visual medial knee displacement	.09 .43 .75	0.5 0.3 0.1
Nguyen et al (2015) [40]	*Femoral anteversion*	15.3(5.2), 9.7(4.7)	> .87 (SEM not reported)	Double-leg drop jump	Hip flexion angle	.07	0.2
					Hip adduction angle	.34	0.2
					Hip IR angle	.16	0.2
					Knee flexion angle	.37	0.4
					Knee valgus angle	.02	0.4
					Knee ER angle	.45	0.1
					Hip flexion moment	.36	0.2
					Hip adduction moment	.11	0.3
					Hip IR moment	.07	0.3
					Knee flexion moment	.46	0.1
					Knee valgus moment	.02	0.4
					Knee ER moment	.002	0.6
Nguyen et al. (2011) [38]	*Femoral anteversion*	10.7(5.2)	> .87(SEM not reported)	Single-leg squat	Hip internal rotation excursion	.06	0.5
					Knee valgus excursion	.82	0.1
					Knee external rotation excursion	.006	0.8
Rabin & Kozol (2010) [44]	*Internal rotation ROM* *External rotation ROM*	41.1(7.9), 48.4(7.4) 62.2(7.4),63.0(4.2)	.91(SEM not reported) .82(SEM not reported)	Lateral step-down	5-point 2D visual criteria (“arm strategy, trunk alignment, pelvis plane, knee position, steady stance”)	.03 .77	1.0 0.1
Stiffler et al. (2015) [47]	Internal rotation ROM External rotation ROM Femoral anteversion	31.9(11.2), 34.2(11.0) 46.1(14.3), 43.3(12.6) 9.7(6.8), 9.4(5.4)	Not reported	Double-leg jump landing	2D medial knee displacement	.61 .25 .81	0.1 0.2 0.2

Note: *IC* initial contact, *PS* post-contact, *ms* milliseconds, *IR* internal rotation, *ER* external rotation

##### Biomechanical 3D results

Five studies [19, 20, 26, 38, 40] examined the relationship between femoral anteversion and three-dimensional valgus measured as initial contact joint angle [26], peak joint angle [19, 20, 26, 40], peak joint moment [19, 20, 40] and/or joint excursions [38] using a single-leg jump landing [26], single-leg forward landing [19, 20], single-leg squat [38], or double-leg drop jump [40]. Due to varying functional tasks, a meta-analysis was not possible. However, generally, greater femoral anteversion was associated with greater initial and peak hip flexion (*d* range = 0.2 – 1.6) [26], peak knee valgus angle (*d* range = 0.1 – 1.5) [19, 20, 26, 40], peak hip internal rotation moment (*d* range = 0.2–0.9) [19, 20, 40], and peak hip internal rotation angle (*d* range = 0.2 – 0.7) [19, 20, 40]. Four studies [9, 19–21] examined the relationship between hip ROM and 3D biomechanics, and generally revealed that greater passive internal rotation was predictive of greater frontal plane hip and knee excursions (*d* range = 0.7–1.2) [21] and frontal and transverse plane knee moments (*d* range = 0.1–1.0) [19, 20].

#### Evidence for a sex-specific effect

Where available, data were stratified by sex and reported in Table 7. Eight injury outcome studies reported sex-specific data (130 females, 670 males). From these studies, ROM differences between injured and uninjured females could be computed in two studies, and between injured and uninjured males in 6 studies. In females, there were no significant differences in passive internal (MD = 1.60°; *p* = 0.68) or external (MD = 1.10°; *p* = 0.33) rotation between ACL-injured and non-injured females [12, 49]. Similarly, in males, there were no significant differences in internal (MD = 3.75°; *p* = 0.09) or external (MD = 1.99°; *p* = 0.22) rotation between ACL-injured and non-injured males [3, 11, 16, 32, 42, 49]. Only one of the 8 studies included both males and females [49], and restricted internal and external rotation was associated with greater injury occurrence only in females.

**Table 7:** Results detailing sex differences in transverse plane femoral alignment in both injury and biomechanical studies

*Injury Outcome*	Study (year)	Clinical Measure	Injured/High Risk Mean (SD)	Uninjured/Low Risk Mean (SD)	*P* value	Cohen’s d
Females	Daneshmandi (2012) [12]	Femoral anteversion Internal rotation ROM External rotation ROM	17.4(5.5) 40.9(5.8) 36.4(3.8)	16.0(6.7) 39.2(6.0) 37.2(3.9)	.46 .38 .51	0.2 0.3 0.2
	Kramer (2007) [28]	Femoral anteversion	10.2(3.0)	11.1(2.7)	.06	0.3
	VandenBerg (2017) [49]	Internal rotation ROM External rotation ROM	24.1(8.2) 57.6(8.4)	30.4(10.4) 42.2(10.7)	.003 < .001	0.7 1.6
Males	Bedi (2016) [3]	Internal rotation ROM	23.4(7.6)	24.5(6.5)	.36	0.2
	Budinski (2016) [11]	Internal rotation ROM External rotation ROM	30.9(11.6) 39.8(11.5)	30.9(10.8) 40.2(11.1)	> .99 .85	0.0 0.0
	Gomes (2008) [16]	Internal rotation ROM External rotation ROM	26.4(7.7) 42.1(9.3)	39.0(7.1) 43.3(8.3)	.001 .48	1.7 0.1
	Lopes (2016) [31]	Internal rotation ROM External rotation ROM	28.6(5.7) 37.5(4.3)	35.6(5.7) 43.7(6.6)	.001 .001	1.2 1.1
	Lopes (2017) [32]	Internal rotation ROM External rotation ROM	33.4(6.4) 41.1(5.8)	32.9(6.0) 40.2(6.4)	.67 .50	0.1 0.1
	VandenBerg (2017) [49]	Internal rotation ROM External rotation ROM	23.4(7.6) 36.9(8.4)	25.1(7.9) 40.2(10.7)	.09 .41	0.2 0.3
*Biomechanical Outcome*						
Females	Bittencourt (2012) [6] jump landing	Internal rotation ROM	51.1(7.6)	44.7(11.2)	.08	0.7
	Bittencourt (2012) [6] single-leg squat	Internal rotation ROM	40.9(11.6)	42.9(12.1)	.56	0.2
	Hogg (2019) [20]	Femoral anteversion Internal rotation ROM External rotation ROM	10.3(5.8) 31.3(8.4) 44.6(9.8)	NA	.07-.92 .05-.68 .09-.85	0.0–0.9 0.2–1.0 0.1–0.9
	Hogg (2021) [19]	Femoral anteversion Internal rotation ROM External rotation ROM	9.4(4.5) 30.4(10.3) 47.8(7.6)	NA	.33-.90 .03-.69 .02-.42	0.0–0.3 0.1–0.7 0.3–0.8
	Kaneko (2013) [26]	Femoral anteversion	20.7(3.3)	16.1(1.7)	.01–1.0	0.0–1.7
	Rabin & Kozol (2010) [44]	Internal rotation ROM External rotation ROM	41.1(7.9) 62.2(7.4)	48.4(7.4) 63.0(4.2)	.03 .77	1.0 0.1
Males	Bittencourt (2012) [6] jump landing	Internal rotation ROM	44.8(12.7)	44.5(18.4)	.95	0.0
	Bittencourt (2012) [6] single-leg squat	Internal rotation ROM	41.9(13.8)	48.3(16.5)	.04	0.4
	Hogg (2021) [19]	Femoral anteversion Internal rotation ROM External rotation ROM	3.0(3.5) 19.8(8.5) 49.6(6.4)	NA	.03-.94 .02-.95 .01-.62	0.0–0.7 0.0–0.8 0.2–0.9

Sex-specific data were available for four biomechanical studies, including 189 females and 220 males [6, 19, 20, 26, 44]. Only one within study biomechanical sex comparison was possible [6]. During a jump landing, females displaying risky biomechanics (high frontal plane knee projection angle) trended toward greater passive internal rotation ROM than females displaying safer biomechanics, but this was not significant (*p* = 0.08, *d* = 0.7). This trend was not observed in the corresponding male cohort (*p* = 0.95, *d* = 0.0). Conversely, during a single-leg squat, there was no difference in internal rotation ROM between females displaying greater or fewer frontal plane knee projection angles, while males who had 6.4° *less* internal rotation ROM displayed more risky frontal plane knee movement (*p* = 0.04, *d* = 0.4) [6].

## Discussion

Our hypotheses were not supported. Although a relationship was observed between hip ROM and ACL injury, the direction of the relationship was contrary to our hypothesis. Specifically, ACL-injured individuals had approximately 5° less available passive internal rotation (*p* = 0.01). Given these findings, and the trend toward ACL injured also having 3° less passive external rotation (*p* = 0.09) than healthy participants, this evidence may suggest that tighter hips are problematic in terms of noncontact ACL injury risk. In regards to our second research question, greater femoral anteversion and passive internal rotation are more often than not associated with frontal and transverse plane hip and knee indicators of dynamic valgus, but there were not sufficient data to conduct meta-analyses with biomechanical studies. With regards to our third research question, though the existence of a sex-specific ACL injury mechanism was not supported with this systematic review, the different trends noted in ROM patterns with both knee biomechanics and ACL injury (less range of motion in males, greater range of motion in females) warrant further investigation. Specifically, fewer degrees of passive ROM in males associated with both risky biomechanics and injury; whereas in females greater passive internal rotation associated with risky biomechanics but not with injury.

### Passive hip ROM and ACL injury

The most salient finding of this meta-analysis was that participants with ACL injuries had 5.02° less internal rotation ROM than did healthy control participants (979 males, 178 females included). This finding was contrary to our hypothesis that *greater* passive internal rotation would predispose one to higher ACL injury risk; however, this is consistent with a related systematic review that investigated healthy and injured ROM differences in vivo and in vitro [10]. Our findings are also consistent with cadaveric research [8], which demonstrated that limited hip internal rotation predicted higher cumulative ACL strain. They theorized that a restriction of hip internal rotation would necessitate a compensatory increase in internal tibial movement in order to complete the desired movement outcome. Greater internal tibial movement, in turn, has consistently been shown to increase in situ ACL strain [15, 29, 33, 36]. This may also explain the trend toward lesser passive hip external in ACL injured (thus greater total hip restriction), which may further exacerbate tibiofemoral joint compensations. Though the link between lesser hip ROM and risky biomechanics is admittedly speculative, having less available passive internal rotation may correspond with one’s ability to generate sufficient hip external rotation torque. Deficits in hip external rotation moments have been demonstrated to put one at risk of ACL injury [45], possibly related to a weak gluteus maximus. Because the gluteus maximus both externally rotates and extends the hip, a weak gluteus maximus would result in poor eccentric control of hip internal rotation *and* hip flexion. Decreased hip flexion during landing is hypothesized to be an ACL injury mechanism [22]. Further research is warranted to determine the efficacy of gluteal strengthening to improve eccentric control of both hip flexion and internal rotation.

Despite the statistical significance of the meta-analyses, the high heterogeneity of the included literature should caution readers against broad application of these findings. Among the six studies detailing the association between passive internal rotation and ACL injury, the observed I^2^ was 92%. For the five studies analyzing passive external rotation and ACL injury, I^2^ was 90%. This means that 92% and 90% of the between-study variation cannot be attributed to chance, but instead are the results of underlying differences in study design and characteristics. Study quality could possibly have contributed to the observed heterogeneity. Of the ten studies comparing either internal or external rotation in ACL-injured and healthy participants, seven of them did not report reliability statistics for ROM measurement (Downs & Black Question 20), resulting in the lowest Downs & Black Quality Index scores. Of the three studies with Quality Index scores above ten, Tainaka (2014) [48] reported the most extreme mean differences for internal and external rotation between ACL-injured and healthy participants. Interestingly, the participants in Tainaka’s study were 13–17 years old, which is younger than the participants in all other studies. Also, passive ROM measures were obtained within several weeks of the initial injury, which was quicker than measurements obtained in all other studies. This suggests that a young adolescent population may possess problematic restricted ROM, or that the ROM limits observed in the initial weeks following injury are different than the ROM limits later in the post-acute phase. High-quality corresponding data in mature populations are lacking, and thus support further research to determine the effect of hip ROM on injury risk across the life span.

Prospective data on this topic is lacking, and retrospective data is limited. One critique of retrospective data is that ROM could be altered as a result of ACL injury, not precede it. Of note, Lopes (2016) compared non-contact ACL injured patients with contact ACL injuries. If ROM changes as a result of ACL injury it follows that the two ACL groups display similar ROM patterns. However, that was not the case. The non-contact injured patients were reported to have 7.0 and 6.2 fewer internal and external rotation degrees of motion than patients injured in with a contact mechanism [31]. This further supports the argument of restricted hip ROM contributing to non-contact ACL injury.

### Passive hip ROM, femoral anteversion, and biomechanics

Due to methodological differences between the biomechanical studies, it was not appropriate to conduct any meta-analyses. Twelve studies were retrieved to assess the relationship between hip ROM and femoral anteversion and biomechanics during a functional task and they varied greatly in the type of biomechanical measure (e.g. 2D vs 3D) and task (e.g. single vs double leg; squat, jumping, cutting). Five of these [4, 6, 35, 44, 47] used 2D criteria to assess biomechanics, while the remaining seven [9, 19–21, 26, 38, 40] used 3D motion capture. A variety of functional tasks were used, including an overhead squat [4], single-leg jump landing [19–21, 26], single-leg squat [6, 35], double-leg drop jump [40], double-leg jump landing [47], a lateral step-down [44], and a diagonal side cut [9]. Seven studies examined the influence of femoral anteversion on biomechanics [19, 20, 26, 35, 38, 40, 47]; eight studies analyzed internal rotation ROM [6, 9, 19–21, 35, 44, 47]; eight studies analyzed external rotation ROM [4, 9, 19–21, 35, 44, 45].

Generally, participants with greater passive external rotation tended to display approximately 4.77° greater 2D medial knee displacement [4, 35, 47]. Not only is this contrary to our hypothesis that lesser passive external rotation would predict biomechanics consistent with functional valgus collapse, but it is also contrary to the injury outcome results which suggested that ACL-injured individuals have less passive external rotation (or less ROM in general) [31, 48, 49]. The disparity between the injury results and the biomechanical results may partly be explained by the distribution of males and females in each set of evidence. The retrospective ACL injury body of evidence included 979 males and 244 females, while the biomechanical body of evidence included 375 males and 452 females. Thus, greater passive external rotation is associated with medial knee displacement in a predominately female cohort. However, it is also possible that the relationship between hip ROM and ACL injury is not mediated by functional knee valgus.

Howard (2011) [21], Hogg (2019) [20], and Hogg (2021) [19] examined relationships between 3-dimensional biomechanics and passive hip ROM. While the results pertaining to external rotation ROM were equivocal, each study demonstrated greater internal rotation ROM to be associated with isolated joint angles and moments consistent with dynamic knee valgus in females. Again, this is contrary to the injury outcome results, and further speaks to the possibility that passive hip ROM influences ACL injury risk differently between sexes, and may influence injury risk in a way that does not act through knee valgus. Females are known to have greater hip internal rotation motion than males, and it is feasible that this greater amount of passive internal rotation may alter lower extremity biomechanics along the kinetic chain differently than males. More research is needed that directly compares males and females on their anatomical hip characteristics and the combined impact on knee biomechanics and ACL injury risk in order to fully understand these relationships and determine a potential mediating effect of knee valgus.

### Sex-specific differences

Within study sex-specific data is lacking, particularly within-study biomechanical data. Regarding ACL injury, the data suggest that in males, limited internal rotation is associated with ACL injury. This is partially supported by the scant biomechanical data available in a male cohort. During a single-leg squat, Bittencourt (2012) [6] observed that males who displayed more risky biomechanics also had less passive internal rotation, though this observation did not hold true for a jump landing in the same cohort. Hogg (2021) [19] used a single-leg landing, reporting males with greater internal rotation ROM display more risky biomechanics. Thus, there may be a task-specific effect, in addition to a sex-specific effect. It has been suggested that males injure their ACLs during sagittal plane mechanisms, instead of during frontal and transverse plane activities [43]. A single-leg squat is a purely sagittal plane motion, while a jump landing challenges frontal and transverse plane control. Conversely, in females, the relationship between passive hip ROM and ACL injury was unremarkable. During a jump-landing, females possessing greater internal rotation ROM displayed more risky biomechanics than females with more limited ROM [6]. When the same cohort performed a single-leg squat, there were no ROM differences between the group identified as high-risk and the low-risk group. This data is far from conclusive and further research is needed to determine if the biomechanical differences observed within each sex are predictive of future ACL injury.

There are limitations of the current systematic review and meta-analysis. Firstly, it was not prospectively registered. Additionally, varying methodology between studies (e.g., 2D v. 3D measurement, choice of functional task) resulted in high observed heterogeneity, which limits the generalizability of our findings. Lastly, more studies are needed to conclusively answer our research questions.

## Conclusion

In conclusion, there is not enough evidence currently available to conclusively determine the effects of femoral anteversion and passive hip ROM on ACL injury and biomechanics. Additionally, there are not enough data to support sex-specific injury mechanisms. Although our meta-analysis indicates that individuals with injured ACLs have statistically less passive internal rotation, the observed heterogeneity compromises the generalizability of our findings. This is contradicted by cross-sectional biomechanical studies suggesting that greater internal rotation ROM, not less, is more likely to be associated with the high-risk biomechanics thought to be associated with ACL injury risk. Specifically, well designed studies including both sexes, and using reliable methods and consistent methodology are needed to investigate biomechanical patterns associated with ROM values, and their prospective associations with ACL injury.

## Supplementary Information

**Additional file 1.** Disclosure of potential conflicts of interest (Jennifer A Hogg)

**Additional file 2.** Disclosure of potential conflicts of interest (Justin Waxman)

**Additional file 3.** Disclosure of potential conflicts of interest (Sandra J Shultz)
